# HAPTI-Child: a finger-tracking video and keypoint dataset of haptic picture exploration and identification in congenitally blind and visually impaired children

**DOI:** 10.3389/fpsyg.2026.1759540

**Published:** 2026-04-23

**Authors:** Dana Wymark, Raymond Roy, Mysa Myers, Oleg Jankech, Gabriel Byczynski, Alfonso Abizaid, Birgitta Dresp-Langley, Amedeo D'Angiulli

**Affiliations:** 1 Neuroscience of Imagination, Cognition, and Emotion Research Lab, Department of Neuroscience, Carleton University, Ottawa, ON, Canada; 2 Department of Neuroscience, Carleton University, Ottawa, ON, Canada; 3 Department of Clinical Neurosciences, University of Geneva, Geneva, Switzerland; 4 Department of Clinical Neurosciences, Geneva University Hospitals, Geneva, Switzerland; 5 Centre National de la Recherche Scientifique, UMR 7357, Strasbourg University, Strasbourg, France

**Keywords:** congenital total blindness, DeepLabCut, finger-tracking video, haptic (tactile) exploration, haptic (tactile) perception, low vision, severe visual impairment, video clips dataset

## 1 Introduction

The study of haptic exploration in childhood blindness offers a clear window into how the developing brain self-organizes when vision provides little or no input on learning and recognition. Early sensory deprivation is associated with cross-modal plasticity, with occipital regions recruited during auditory and tactile processing (Lewis et al., 2010; Pang et al., 2024; Rabinowitch and Bai, 2016); such reorganization furnishes a natural testbed for self-organization in development, including modular coordination, local-to-global organization, resilience, growth, and adaptive plasticity (Dresp-Langley, 2020). The HAPTI-Child dataset contributes to this agenda by capturing naturalistic raised-line picture identification in congenitally totally blind (CTB) and severely visually impaired (SVI) children without corrective feedback, allowing exploratory strategies to unfold under conditions that closely resemble everyday tactile learning.

Work in tactile graphics and haptic perception has long emphasized that blind and SVI individuals actively construct tactile representations through exploration rather than merely registering input passively. Mazella, Picard and colleagues developed a multi-component haptic test battery for children and adolescents with and without visual impairment (Mazella et al., 2014), comprising tasks probing shape scanning, discrimination, spatial understanding, tactile short-term memory, and picture comprehension. In a preliminary study with 13 visually impaired and 13 blindfolded sighted children aged 9–10 years, the two groups achieved comparable overall scores; however, visually impaired children responded more quickly and obtained higher accuracy in shape and texture discrimination and in raised-line picture comprehension (Mazella et al., 2014). Converging evidence from tactile picture processing similarly indicates some performance advantage for congenitally blind children relative to blindfolded sighted peers (D'Angiulli et al., 1998), while also demonstrating how practice in guided, systematic exploration mediates picture identification and can partially remediate performance in the blindfolded sighted group (D'Angiulli and Kennedy, 2000).

Developmental trajectories reinforce the idea that tactile picture identification reflects maturing self-organizing representational capacities shaped by practice, memory, and strategy choice rather than a simple consequence of sensory status. In a developmental study with sighted participants aged 5–25 years, blindfolded children aged 5–7 correctly identified about one third of raised-line drawings, whereas accuracy increased to 69 % in adolescents and 86 % in adults, paralleling improvements in haptic short-term memory (Picard et al., 2014). Reviews of tactile picture recognition highlight that identification rates span 9–85 % across studies and depend strongly on age, visual status, haptic practice, availability of semantic information, and the exploratory procedures deployed (Vinter et al., 2020); category cueing prior to exploration can facilitate hypothesis generation, yet visually impaired children often maintain an advantage by combining cueing with more efficient haptic strategies (Mazella et al., 2014). Related results from three-dimensional exploration point toward systematic differences in the dynamics of haptic search, with early blindness associated with distinctive patterns of contact and strategy switching even when recognition accuracy converges across groups (Leo et al., 2022); reports of superior navigation with tactile maps in congenitally blind adults further underscore functional plasticity that emerges through practice and experience (Boumenir et al., 2014).

Across this literature, the most consistent pattern of findings is that haptic picture identification is demanding, developmentally graded, and sensitive to both task constraints and the exploratory procedures adopted (Picard and Lebaz, 2012). At the same time, empirical patterns remain heterogeneous, with performance advantages sometimes favoring visually impaired participants, sometimes blindfolded sighted participants, and sometimes neither group, reflecting differences in stimulus complexity, representational familiarity, and prior haptic experience (Vinter et al., 2020) and working memory/imagery ability (Holtby and D'Angiulli, 2012). Such variability highlights a methodological limitation in much of the existing evidence base, namely, an emphasis on holistic outcomes such as identification accuracy at conclusion of task without an equally detailed record of the hand and finger dynamics through which recognition unfolds; resolving that limitation requires resources that preserve the temporal structure of exploration itself.

Unsupervised learning frameworks such as Self-Organizing Maps (SOM) and Adaptive Resonance Theory (ART) posit that local interactions can yield stable global representations, yet key empirical gaps persist in the behavioral record needed to evaluate such accounts. Many benchmarks remain abstract or heavily preprocessed, obscuring the embodied, sequential nature of real-time exploration (Kriegeskorte and Douglas, 2018); sequential strategy formation remains under-constrained because available resources rarely include time-resolved motor trajectories capturing how fingertips, digits, and hands coordinate over hundreds to thousands of milliseconds (Mueller et al., 2019). Moreover, while neural self-organization, as stipulated in ART, addresses the stability-plasticity problem, few developmental datasets support quantitative tests of how new categories of representations are integrated without catastrophic interference under child-level learning conditions (Grossberg, 2013); these limitations restrict attempts to connect theoretical predictions about modular coordination and adaptive flexibility to perceptual behavior when vision is absent.

To offer tools that might help addressing these research gaps, we built HAPTI-Child. Originated in the context of a previous study (D'Angiulli et al., 2024), the present resource was compiled retrospectively to include trial-segmented videos, labeled finger and hand keypoints produced with DeepLabCut, trained pose-estimation models, Colab analysis notebooks, and derived per-frame kinematic measures across thousands of frames for children recorded during raised-line picture identification. The time-resolved trajectories support the investigation of modular coordination within and across hands and enable analyses of how local exploratory movements aggregate into global recognition strategies over time, while the combination of naturalistic behavior and shared task constraints offers a tractable benchmark for supporting the development of unsupervised models. Researchers can explore how self-organization modeled by SOM or ART algorithms may capture local-to-global transitions, age-dependent adaptations, and strategy stabilization as new exemplars accumulate, exploring possible types and levels of correspondence between computational claims and measurable motor structure.

The dataset also speaks to open questions in cross-modal plasticity, where behavioral signatures of exploration may provide an interpretable bridge between sensory reorganization and functional outcomes. Such questions align with broader perspectives on multisensory integration and embodied interaction in technologically mediated contexts (Cornelio et al., 2021). If exploration strategies in blind children are intertwined with recruitment of brain visual areas (e.g., occipital cortices), as suggested by stimulus-responsive and task-dependent activations in early blindness, then linking detailed movement signatures to performance becomes a critical step toward connecting behavior with reorganization in visual cortex (Lewis et al., 2010; Pang et al., 2024; van der Heijden et al., 2019). In parallel, our dataset can be combined with companion work on verbal mediation in blindness to examine how speech may scaffold or reshape exploratory strategy formation in development (see D'Angiulli et al., 2024); although HAPTI-Child does not include direct neuropsychological assessments of executive control, the fine-grained hand- and finger-tracking data provide behavioral proxies for attentional allocation (e.g., dwell-time on diagnostic features or the frequency of strategy switches), which can be paired with standardized executive function measures in future studies to clarify how attentional control shapes haptic exploration.

Beyond theory testing, HAPTI-Child has translational implications, since fine-grained descriptions of successful vs. inefficient strategies can inform haptic training protocols and sensory-substitution design while also offering structure for bio-inspired AI systems that learn robust concepts from touch under minimal supervision. This paper introduces the dataset, details acquisition and processing methods, reports technical validation and usage notes, and outlines applications for modeling and developmental research, positioning HAPTI-Child as a resource for advancing unsupervised learning theories with developmentally grounded evidence and for clarifying how adaptive organization may support perception without vision.

## 2 Methods—data collection and processing

### 2.1 Participants

Sixteen children participated: nine CTB; no residual vision and seven SVI. To standardize sensory input across groups, SVI participants were blindfolded during the tasks. Blindfolding ensured that both groups relied exclusively on haptic information when exploring raised-line drawings, enabling meaningful comparisons of exploration strategies. In addition, both blind and blindfolded sighted participants yield generally low accuracy rates at naming raw raised-line drawings. Using a blindfolded group helps researchers identify the “floor-level”, when a task is inherently too difficult when relying on tactile and haptic inputs alone, rather than a specific deficit in the blind group (see Heller, 2002). However, removing residual even minimal visual cues may have disadvantaged the SVI participants relative to their everyday exploratory habits; this limitation should be considered when interpreting group differences or should be included as variable (i.e., covariate) in the analysis and research questions.

Inclusion criteria were no additional neurological or psychiatric diagnoses and ability to assent. Demographic and clinical characteristics recorded for each participant included sex, age (reported in “years: months”), visual classification (congenitally totally blind or low vision with specified visual acuity), and underlying cause of visual impairment. All demographic data are available in the following zenodo repository https://doi.org/10.5281/zenodo.18930624. The two groups were matched in terms of SES, age and sex, and Braille proficiency distributions (for further details, see Footnote in Demographic information Tables at https://doi.org/10.5281/zenodo.18930624), they were also within the same school mixed-grade cluster according to the Montessori system (for a complete description of the samples and their matching please refer to D'Angiulli et al., 2024). Recruiting CTB and SVI children is challenging, and the present sample reflects both the rarity of the samples and the resource-intensive nature of acquiring and annotating high-resolution haptic videos. As such, the dataset should not be viewed as a statistical sample for population-level inference. Instead, it provides richly annotated examples for benchmarking unsupervised sequence-learning and strategy-analysis methods. Nevertheless, users should exercise caution when drawing group-level conclusions and treat the data primarily as a platform for hypothesis generation and model development. We also stress that the use of this database should be supplemented with other additional and external experimental observations and sources of information.

The protocol was approved by the ethics research boards of the Italian National Institute for the Blind (Unione Italiana Ciechi), the Universita' degli Studi di Padova (University of Padua) and Carleton University. All procedures adhered to ethical principles for research with minors as set by Canada's Tri-Council Policy Statement: Ethical Conduct for Research Involving Humans. Written parental consent and child assent were a requirement for participation and included permissions for publishing and sharing de-identified derivatives. Conditions for raw-video access are specified in Section 3 and Section 6 Conclusion and Future Directions.

### 2.2 Stimuli and apparatus

#### 2.2.1 Tactile stimuli

The raised-line drawings were made by tracing ink pictures with a stylus producing contact relief traces (~ 0.5 mm high) on transparent Mylar plastic sheets that were 21.6 cm × 27.9 cm in size; the objects depicted subtended an imaginary rectangular area of up to maximum 16.5 cm and 14.5 cm in the *Y* and *X* dimensions, respectively. Preliminary analysis showed that neither the single dimensions nor areas of these stimuli were significantly correlated with identification accuracy or response latency. Response latency was defined as the elapsed time between the participant's first hand contact with the stimulus and the vocalization of their final verbal identification response (or a no-label response), such that for each trial *i*:


$$\begin{array}{l} L_{i}=t_{final-response\;i}{\;-\;t}_{first-contact\;i} \end{array}$$

This measure therefore includes the time required for tactile exploration as well as decision, response selection and verbalization processes. A thumbnail sheet with the ID key accompanies the dataset for consistent referencing.

#### 2.2.2 Recording setup

Data collection was conducted using a Toshiba SK-F200 VHS camcorder. The camera was set up on a fixed overhead mount which permitted to record the work surface and arms and hands under uniform lighting; audio captured open-ended responses and experimenter prompts. Camera, lens, codec, and microphone specifications are recorded in per-session metadata (full make/model fields documented in the data dictionary).

### 2.3 Procedure

The experiment consisted of five sequential phases administered individually to each participant.

Phase 1: Familiarization Practice

To ensure comprehension of the task and comfort with tactile exploration procedures, children were first given the option to complete a brief practice phase. Six raised-line drawings were available for practice (Apple, Cane, Hand, Hanger, Fork/Spoon, and Shoe). Participants who opted to complete this practice phase were given an image at random and could complete the phase with as many of the images (up to six times) as they desired. For each selected image, participants freely explored the drawing and provided an open-ended identification. No corrective feedback was provided. This phase was intended solely to familiarize participants with the exploration and response format and was not included in subsequent analyses.

Phase 2: Core Identification Task (Test)

Participants then completed the primary exploration–identification task using a fixed set of 10 core raised-line drawings: Figure, Key, Face, Umbrella, Leaf, Scissors, Bottle, Mug, Phone, and Lamp (the visual ink blueprints of these pictures are available at https://doi.org/10.5281/zenodo.18930624). Stimuli were presented in a participant-specific randomized order. For each image, children were instructed to explore the drawing and verbally identify what it depicted (e.g., “This is a picture of ___?”). Participants were also asked to justify their responses (e.g., “What made you think that?”). No corrective feedback was provided at any point. Sighted visually impaired participants completed all tasks blindfolded. This phase constituted the first identification run (test).

Phase 3: Intervening Drawing Task

Following the initial identification run, participants completed an intervening drawing task. Children were invited to select one or two of the core stimuli they had not successfully identified and to produce a drawing reflecting how they understood the tactile image. During this phase, participants were permitted to re-explore the selected stimuli while drawing. This activity was designed to: (a) support encoding and consolidation of the tactile information and (b) capture the participant's internal representation of the stimulus relative to its tactile configuration. No feedback was provided. Because the focus of our present study was on the core exploratory phases of this experiment, the drawing phase was omitted from our analyses.

Phase 4: Core Re-Test

Participants then completed a second identification run (re-test) using the same 10 core stimuli (Figure, Key, Face, Umbrella, Leaf, Scissors, Bottle, Mug, Phone, and Lamp). Stimuli were again presented in randomized order. Procedures and instructions were identical to those used in Phase 2, and no corrective feedback was provided. This phase enabled assessment of changes in identification performance across repeated exposure.

Phase 5: Optional Free Exploration

At the conclusion of the experimental phases, participants were offered the opportunity to explore additional stimuli from the practice set for enjoyment. This phase was optional and was not included in formal analyses.

Data Discrepancy

Although the experimental protocol comprised 10 primary haptic stimuli administered in a repeated test–re-test format, complete video documentation was not available for every participant–stimulus pairing. We acknowledge this discrepancy and note that it reflects practical constraints rather than systematic procedural inconsistencies. As mentioned, data was collected by using a VHS camcorder, with recordings archived on analog VHS tapes. During long-term storage and subsequent handling for digitization, portions of several tapes exhibited signal degradation and corruption. In these instances, specific segments could not be reliably extracted despite multiple transfer attempts, resulting in isolated gaps in the archival record.

In addition, a small proportion of participants did not complete all 10 trials. Given the young age of the sample and the attentional and sensory demands of repeated haptic exploration without feedback, some children exhibited mental or physical fatigue and discontinued participation before task completion. These factors collectively account for the minor inconsistencies in available video data across participants and stimuli and reflect logistical and developmental realities inherent to research with pediatric populations rather than selective data loss.

As result of the data collection and storage challenges, the final corpus of data included 275 stimulus-video-clip shot triads: 259 from the core identification tests and re-tests, and 16 from both the first optional practice phase and the last optional exploratory phase. Overall data loss from the first core identification test was calculated as 13.8%, while in re-test, data loss was calculated as 24.4%. For transparency, we have also included a table outlining the data recorded from the main test phases of the experiment in the document *detailed description of database contents*, available at https://doi.org/10.5281/zenodo.18944507.

#### 2.3.1 Timing definitions

Trials were self-paced. Onset (first contact) was established by the first-hand contact with the sheet; offset (final response) was determined by the vocalization of the final verbal label (or a no-label response). Inter-trial intervals and break durations were logged.

#### 2.3.2 Randomization

A per-participant permutation of S01–S13 was sampled for each run; the random seed is stored in session metadata.

### 2.4 Segmentation and anonymization

Raw session videos were split into trial-level clips and organized by participant, run, and stimulus. A sidecar JSON captures participant ID, group, age, sex, hand use, run, stimulus ID, trial index, trial start/end timestamps, camera specs, and any protocol deviations. Framing excluded faces; incidental identifiers were cropped or redacted. Audio was retained for scientific content; any personal names were bleeped with time-stamped redaction logs.

### 2.5 Keypoint labeling and model training

#### 2.5.1 Tooling

Markerless tracking used DeepLabCut (DLC) in GPU environments. >6,000 frames were manually labeled across diverse hand poses and stimuli following standardized guidelines (included in the repository); they were, for each hand, center of palm, thumb, and index to pinky. Accordingly, 12 total key points were defined.

#### 2.5.2 Training and evaluation

A ResNet-backbone was fine-tuned; the model selection used the lowest validation pixel error. Performance on a held-out test set was summarized by mean/median pixel error, with millimeters calibration and percentage-of-correct-keypoints (PCK) at multiple thresholds. Inference produced per-frame (*x, y*, likelihood) for all keypoints. Low-confidence points were filtered by a fixed likelihood threshold; short gaps were interpolated (see Section 2.6). Trained weights and configurations were archived with checksums.

#### 2.5.3 Additional calibration

When a printed grid was visible, pixel-to-mm conversion and its uncertainty are recorded in metadata for optional metric analyses.

### 2.6 Derived measures and visualization pipeline

Processing was implemented in Python (versions were pinned in the environment file) with Pandas/NumPy/Matplotlib. Steps:

- **Filtering and interpolation:** per-frame likelihood filtering; linear interpolation of brief gaps; longer gaps remain missing.
- **Smoothing:** Gaussian Kernel Density Estimation was used for smoothing of spatial density.
- **Kinematics:** measures included fingertip trajectories, inter-finger distances, and dwell time heatmaps over the stimulus plane. Per-trial exploration duration was not explicitly calculated in the available pipeline.
- **Normalization:** coordinate normalization to millimeters was not implemented in the available code. Normalization was limited to optional transformation into stimulus coordinate space.
- **QC views:** trajectory overlays and heatmaps per stimulus/participant (thresholds and criteria in Section 5 Technical Validation).

Versioned notebooks generate all figures and tables from the released CSVs/JSON without manual steps.

## 3 Data records

### 3.1 Repository and DOI

Data are publicly available from the following open access links: https://doi.org/10.5281/zenodo.17359372 (“Version 1”—this link contains all processed video clips) as well as https://doi.org/10.5281/zenodo.18615711 (“Version 2”—this link contains guidelines on how to access the data with an alternative link to Google drive).

## 4 Contents overview

### 4.1 Dataset distinction and format conventions

Following the dataset's DOI link to the repository provides access to the complete file structure and contents of two complementary video sets: CTB_videos and SVI_videos. CTB_videos contains recordings from nine congenitally totally blind participants, and SVI_videos contains recordings from seven sighted visually impaired participants. The two datasets share a common camera setup, timing protocol, and file-naming convention, but differ slightly in the stimulus set.

All recordings are provided in MP4 format and follow a consistent naming scheme: *participant[number][object][run].mp4*. The run index (1 or 2) denotes the first and second trial for each object. Folder organization groups recordings by object type and task iteration, supporting transparent and reproducible navigation across both datasets. Together, CTB_videos and SVI_videos comprise 16 participants across all tasks, representing blind and sighted visually impaired children.

In addition, the Appendix repository contains the demographic information for each of the 16 participants, including subject number, sex, age, blindness severity, and medical cause of visual impairment. The repository also includes scanned images of the 10 stimuli used during the haptic exploration phase: face, bottle, mug, figure, lamp, phone, umbrella, scissors, key, and leaf.

## 5 Technical validation and example of descriptive analyses

Test set performance of the DeepLabCut models (see Section 2.5 for training) was evaluated using standard pixel error metrics (mean ± SD and median ± IQR). Where calibration targets were available, these values were converted to millimeters. In addition, PCK was reported at fixed thresholds, providing a scale-independent indicator of accuracy.

For each keypoint, the proportion of frames above the likelihood cutoff was computed to define trial-level completeness. Frames falling below threshold were marked as missing. Short gaps were interpolated using the preprocessing pipeline described in Section 2.6, while longer gaps remained flagged for transparency. Distributions of missingness and maximum gap lengths were summarized across all participants and trials to guide secondary analyses.

Data quality was further assessed through biologically grounded criteria. Within-trial smoothness was examined by deriving instantaneous velocities and accelerations from central differences; observed ranges were consistent with plausible finger kinematics for children of this age group. Cross-run stability was evaluated by comparing dwell-time maps and clustered trajectories across repeated presentations of the same stimulus for each participant, showing stable coverage patterns.

**Figure 1:**
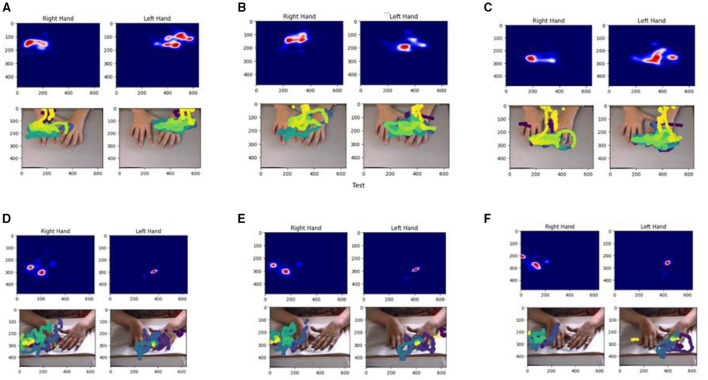
Representative hand- and finger-specific exploration density maps and trajectories. **(A)** Back of hands, **(B)** thumb tips, **(C)** index finger tips, **(D)** middle finger tips, **(E)** ring finger tips, and **(F)** pinky finger tips.

To provide a transparent overview of the dataset's scope without making inferential claims, we generated individual and then aggregated group-level visualizations such as contrasted heatmaps of exploration density and distance-over-time traces. Figure 1 illustrates a representative example from participants, showing right- and left-hand dwell-time heatmaps and trajectory overlays for the back of the hands and each fingertip. All quantitative quality control outputs, including complete distributions and trajectory statistics, as well as visualizations like those in Figure 1, can be regenerated through the shared notebooks provided in the repository.

For a single trial from one participant, panels show right- and left-hand exploration of a raised-line image. Within each column, the top row depicts Gaussian kernel density–smoothed dwell-time heatmaps for a given keypoint class (back of the hands, thumb tips, index fingertips, middle fingertips, ring fingertips, and pinky fingertips). Brighter regions indicate locations visited more frequently during exploration. The corresponding bottom row overlays color-coded fingertip and hand trajectories on the original video frames, illustrating how coverage of the workspace differs across hands and digits. These visualizations are generated automatically by the provided quality-control notebooks and exemplify the per-trial movement summaries available in the HAPTI-Child dataset.

**Figure 2:**
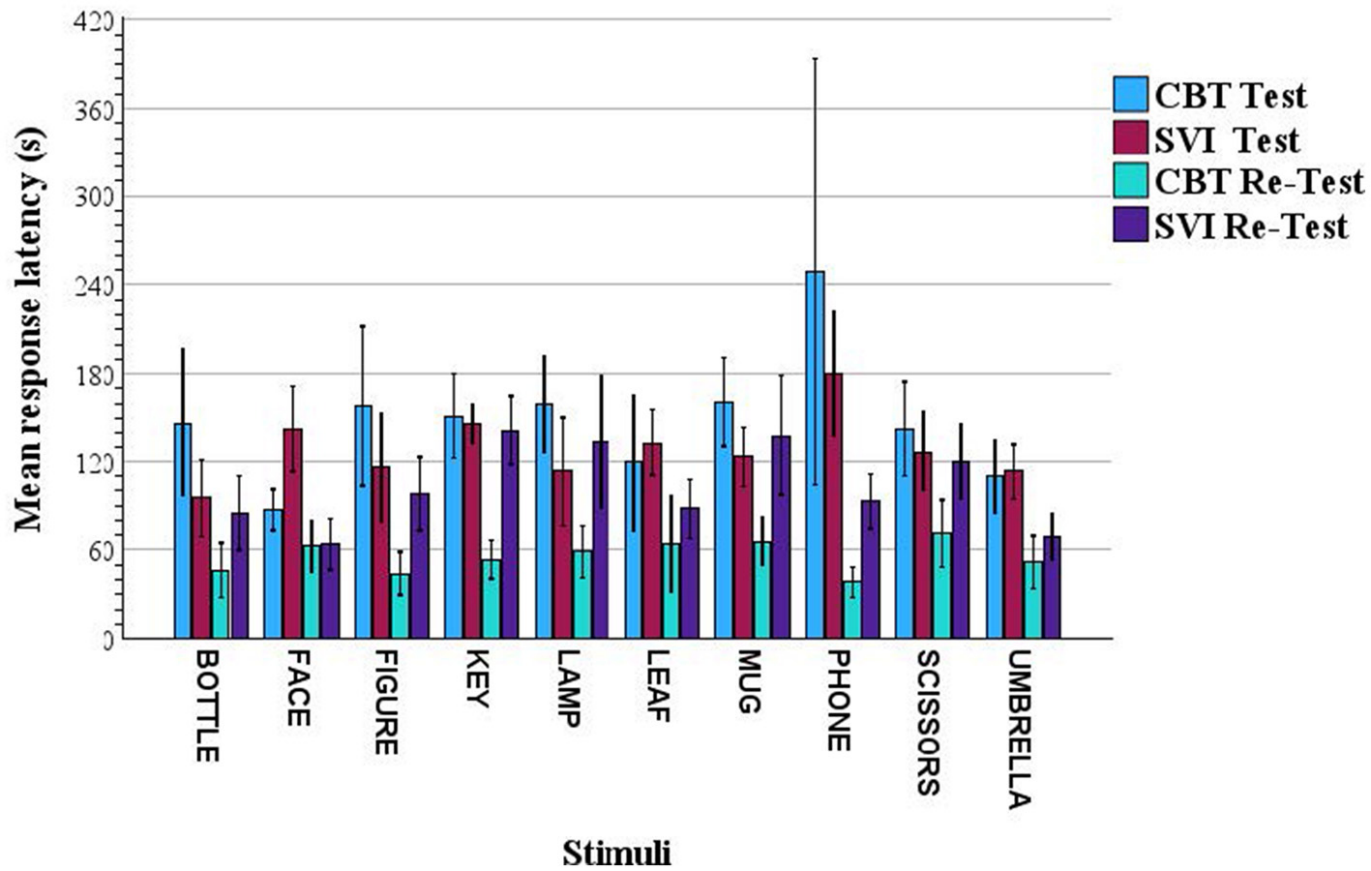
Mean response latencies for each raised-line image by group in test (run 1) and Re-test (run 2) conditions.

As an additional descriptive check on task-level performance patterns, we examined the mean exploration durations for each of the raised-line images across the two participant groups (CTB and SVI). These times were derived directly from trial onset and offset definitions (Section 2.3), averaged across participants within each group. Figure 2 illustrates the resulting profiles. Although the analysis is strictly preliminary and descriptive (not intended as inferential), it provides a compact summary of where the two groups converged or diverged in task demands.

The *y*-axis shows mean response latency in seconds. As defined in Section 2.2.1 (i.e., latency for each trial *i* computed as: *L*_*i*_ = *t*_*final*−*response i*_ − *t*_*first*−*contact i*_, where *t*_*final*−*response i*_corresponds to the moment of initial hand contact with the stimulus and *t*_*first*−*contact i*_ to the vocalization of the participant's final verbal identification response, or a no-label response). This measure therefore includes time of tactile exploration as well as decision, response selection and verbalization processes. Trial-level latencies were averaged within each group, stimulus, and condition.

The *x*-axis lists the raised-line images presented during the identification task. The colored bins represent group means for Congenitally Totally Blind (CTB) and Severely Visually Impaired (SVI) participants. For each group, the bins are color-coded to indicate performance during the Test (phase 2; Core Identification Task) and Re-Test (phase 4) conditions (see legend at top left). Error bars represent ±1 standard error.

It may be assumed that SVI participants retained partial early visual experience that could support the formation of more efficient object representations (e.g., via visual mental imagery) when translated into touch. However, in the test condition there were no differences in response latencies between groups, either at the intercept level or as a function of stimulus identity.

Nevertheless, notable differences seemed to emerge in the re-test condition but did not suggest an advantage for the SVI group. Specifically, for a subset of stimuli (4/10: KEY, FIGURE, MUG, and PHONE), CTB participants showed markedly reduced response latencies during re-test trials. These stimuli contain curved contours and spatially distributed features that often require slower, more deliberate global evaluation, which may have conferred a performance advantage to CTB participants upon repeated exposure. In contrast, the remaining stimuli showed largely overlapping latency distributions across groups. The latter objects are characterized by highly diagnostic linear features that can be rapidly localized, suggesting that both groups may have relied on similar local-feature-based exploration strategies in these cases.

Accuracy in identification rates are traditionally scored for exact correct label naming. Exact label naming shows similar accuracy results around 35 % and 40% for test and re-test accuracy rates in both CTB and SVI groups. These rates are in line with most recent literature (Mazella et al., 2016; Overvliet and Krampe, 2018). Overall, the descriptive patterns indicate that stimulus-specific structure plays a major role in shaping strategy complexity and can reduce or reverse the broader group pattern differences. These observations provide a useful base for future kinematic analyses that can examine movement modularity, dwell-time organization, and transitions across exploratory states. They also highlight the influence of item-level features on the organization of haptic exploration across the dataset.

### 5.1 Interpretability and reuse notes

Below, we outline the main reusability features:

1. Coordinate system and scaling. Trajectories are in image-plane pixels (origin top-left; *x* rightward; *y* downward). Convert to mm using per-session calibration (Section 2.5). Lens distortion was negligible at the working distance; users may apply radial correction if needed.
2. Event timing. Trial onset/offset definitions follow Section 2.3; timestamps align with frame indices and experimenter logs.
3. Preprocessing defaults. Likelihood filtering, interpolation of brief gaps, Gaussian Kernel Density Estimation smoothing, and finite-difference kinematics are implemented in the notebooks (Section 2.6). Parameters are easily adjustable.
4. Starter tasks. (i) SOM-based trajectory clustering to reveal exploratory modules and transitions; (ii) CTB vs. SVI contrasts of movement profiles as potential behavioral correlates of task-dependent cortical recruitment; (iii) optional language–action analyses when paired with companion transcripts.
5. Environment and reproducibility. Python version, package versions, OS, and GPU/CPU details are pinned; notebooks execute end-to-end from released files.

## 6 Conclusion and future directions

HAPTI-Child provides a trial-resolved, publicly reusable record of pediatric tactile exploration that preserves the fine-grained temporal structure through which local finger movements organize into global recognition strategies in the absence of vision. By combining naturalistic task conditions with markerless hand and finger tracking, the dataset captures exploratory behavior at a level of resolution that has been largely absent from prior work on tactile picture identification, while remaining fully reproducible through shared code, pinned environments, and transparent quality-control procedures.

A central contribution of this resource lies in its suitability as a benchmark for methodological development. The time-aligned trajectories and derived kinematic measures enable systematic evaluation of unsupervised and weakly supervised approaches to strategy elaboration, including trajectory clustering, sequence modeling, and representation learning. Because all analyses can be regenerated from the released data using standardized scripts, HAPTI-Child supports direct comparison across modeling frameworks using common readouts, facilitating cumulative progress rather than isolated demonstrations. In parallel, the recorded action trajectories offer a realistic empirical substrate for bio-inspired robotics and assistive technologies, where effective tactile control, grasp exploration, and navigation-in-contact depend on learning from authentic patterns of haptic search rather than idealized inputs.

At the same time, the scope of the dataset is intentionally constrained; collected from a single cohort within one institutional context and focused on raised-line drawings, HAPTI-Child is not designed to support population-level inference or to generalize across all tactile tasks. Its primary value lies instead in hypothesis generation, comparative modeling, and the development of analytic tools under clearly articulated assumptions. Ethical reuse guidelines and controlled access to raw video materials balance participant privacy with the need for open, verifiable science, ensuring that reuse remains both responsible and rigorous.

The descriptive analyses included here illustrate how stimulus structure shapes the demands placed on exploration strategies. Response latency profiles, such as those shown in Figure 2, provide a behavioral anchor for interpreting movement-level measures, revealing how item-specific features modulate efficiency and strategy complexity across groups. These patterns underscore the importance of analyzing exploration as a dynamic process rather than relying solely on endpoint performance, and they motivate future work aimed at formalizing indices of sustained attention, strategy switching, and hemispheric asymmetry reflected in hand-use patterns.

Looking forward, progress in this area will benefit from interoperable resources that extend the task ecology beyond raised-line drawings, incorporate appropriate comparison cohorts, and integrate complementary modalities such as force or pressure signals where ethically and technically feasible. We explicitly invite community contributions in the form of compatible datasets and extensions to the shared analysis notebooks, with an emphasis on reproducibility, transparent preprocessing, and standardized reporting practices that support cross-dataset synthesis.

Finally, HAPTI-Child aligns naturally with emerging theoretical perspectives that emphasize coherence under constraint as a driver of self-organization in biological and artificial systems. In particular, the data set provides a concrete testbed for examining predictions derived from the Precision Principle (Roy et al., 2025), enabling quantitative analyses of how modular coordination and resource efficiency evolve over time as exploratory behavior stabilizes into adaptive strategies. By supporting integration across developmental neuroscience, computational modeling, and bio-inspired robotics, HAPTI-Child offers a foundation for translating detailed behavioral structure into mechanistic insight, while remaining grounded in the empirical realities of tactile learning without vision. Just as a navigator cannot rely on a single star but must consider the configuration of many, coherent recognition emerges not from isolated touches but from the organized coordination of multiple local signals over time.

## Data Availability

The original contributions presented in the study are included in the article/supplementary material, further inquiries can be directed to the corresponding author.
